# A metal–organic framework immobilised iridium pincer complex†
†Electronic supplementary information (ESI) available: Detailed experimental procedures. CCDC 1465323. For ESI and crystallographic data in CIF or other electronic format see DOI: 10.1039/c6sc01376g


**DOI:** 10.1039/c6sc01376g

**Published:** 2016-05-10

**Authors:** Martino Rimoldi, Akitake Nakamura, Nicolaas A. Vermeulen, James J. Henkelis, Anthea K. Blackburn, Joseph T. Hupp, J. Fraser Stoddart, Omar K. Farha

**Affiliations:** a Department of Chemistry , Northwestern University , 2145 Sheridan Road , Evanston , Illinois 60208 , USA . Email: jfstoddart@gmail.com ; Email: omarkfarha@gmail.com; b Department of Chemistry , Faculty of Science , King Abdulaziz University , Jeddah , Saudi Arabia

## Abstract

An iridium pincer complex has been immobilised in the metal–organic framework **NU-1000** using a technique called solvent assisted ligand-incorporation (SALI). The framework proved to be stable under the conditions required to activate the iridium complex and spectroscopic investigations showed formation of the catalytically active iridium dihydride. The Ir-pincer modified **NU-1000** is an active catalyst for the condensed phase hydrogenation of a liquid alkene (1-decene and styrene) and shows enhanced activity with respect to a homogeneous analogue. Additionally, the Ir-pincer immobilised inside **NU-1000** operated as an efficient heterogenous catalyst under flow conditions.

## Abstract 2

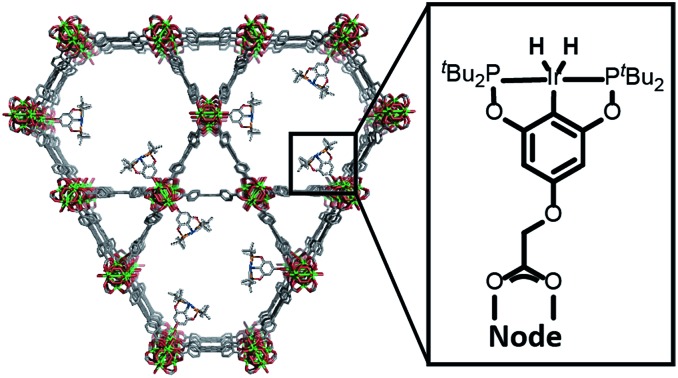
An iridium pincer complex has been immobilised in the metal–organic framework **NU-1000**. The stable Ir-pincer modified **NU-1000** is catalytically active in the hydrogenation of alkenes in condensed phase and under flow conditions.

## Introduction

Pincer complexes have been widely used and investigated for application in homogeneous catalytic transformations.^1^ Iridium pincer complexes are of special interest and were first prepared two decades ago.^2^ Their chemistry is still the object of numerous investigations^3^ and this class of complexes are extremely effective in applications involving hydrocarbon conversions.^4^ Heterogenisation of organometallic complexes has been under extensive study, especially employing amorphous metal oxides as supports.^5^

Generally, supporting an organometallic species requires the use of organic ligands designed to enable anchoring on a heterogeneous substrate.^6^ The ligands are often complex and require a significant investment in synthesis. This approach aims to yield single sites or usually well-defined systems, whose characterisation is not always straightforward.

Recently this approach has also been applied to the post-synthetic metalation of MOFs to afford, amongst others, Co(ii)- and Fe(ii)-based single-site catalysts that were found active in various transformations. In particular, high turnover numbers were obtained in alkene hydrogenations. Catalytic tests where typically conducted at 40 bar, room temperature and using THF as solvent.^7^

To date, only a few heterogeneous materials containing pincer complexes have been prepared and used in catalytic processes.^8^

**Fig. 1:**
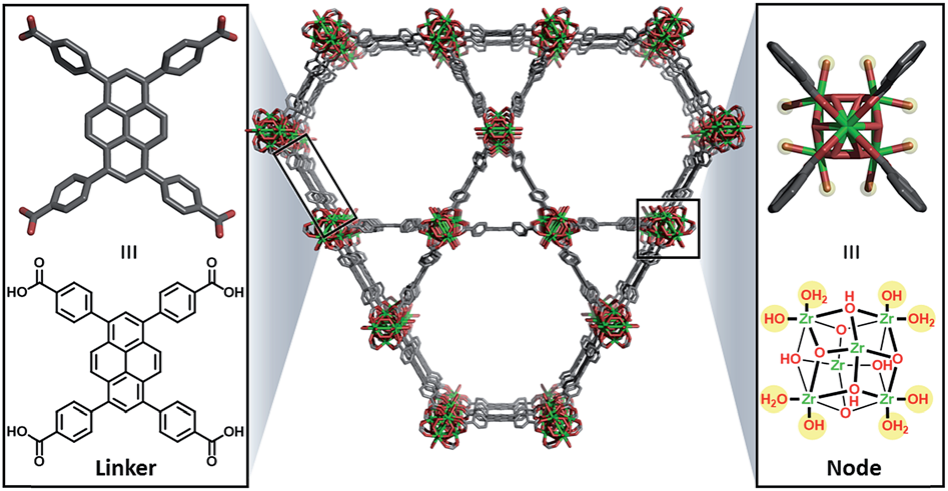
Unfunctionalised **NU-1000** showing the linker (left) and node (right) with the displaceable –OH and H_2_O ligands highlighted in yellow.

Herein, we describe the incorporation of a catalytically active iridium pincer complex into a metal–organic framework (MOF). We selected **NU-1000**^9^ – a mesoporous MOF (Fig. 1) featuring 30 Å channels with Zr_6_ oxide metal nodes and 1,3,6,8,-tetrakis(*p*-benzoic acid)pyrene as the tetratopic linkers—as the heterogenization platform. Detailed physical and structural characterisations, as well as the elucidation of the proton topology around the node, have been described^9^,^10^ recently and a post-synthetic modification, called solvent assisted ligand-incorporation^11^ (SALI) has been used to decorate the metal nodes of **NU-1000** with carboxylic acid containing molecules. SALI makes use of carboxyl groups to displace labile –OH and H_2_O ligands on the Zr_6_ node and form strong chelating metal–ligand bond. In this report, an Ir(iii) complex featuring a bis-phosphinite pincer ligand, similar to those introduced and developed by Brookhart and co-workers,^12^ has been incorporated into **NU-1000** using SALI and its catalytic activity tested in a hydrogenation reaction.

## Results and discussion

**Scheme 1:**
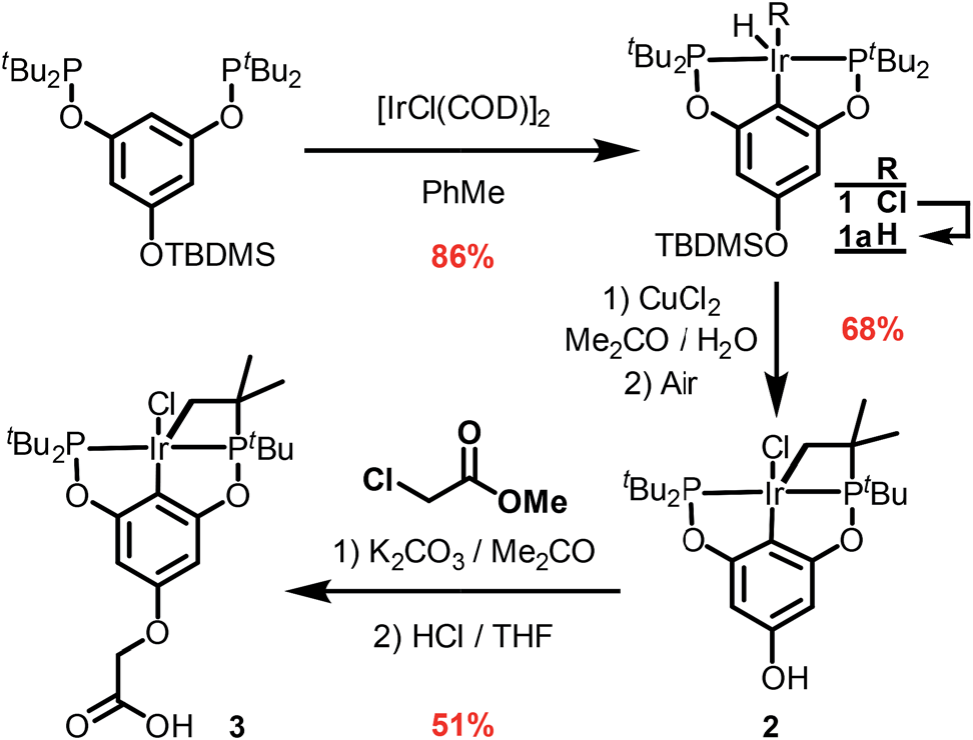
Synthesis of the complex **3**.

**Fig. 2:**
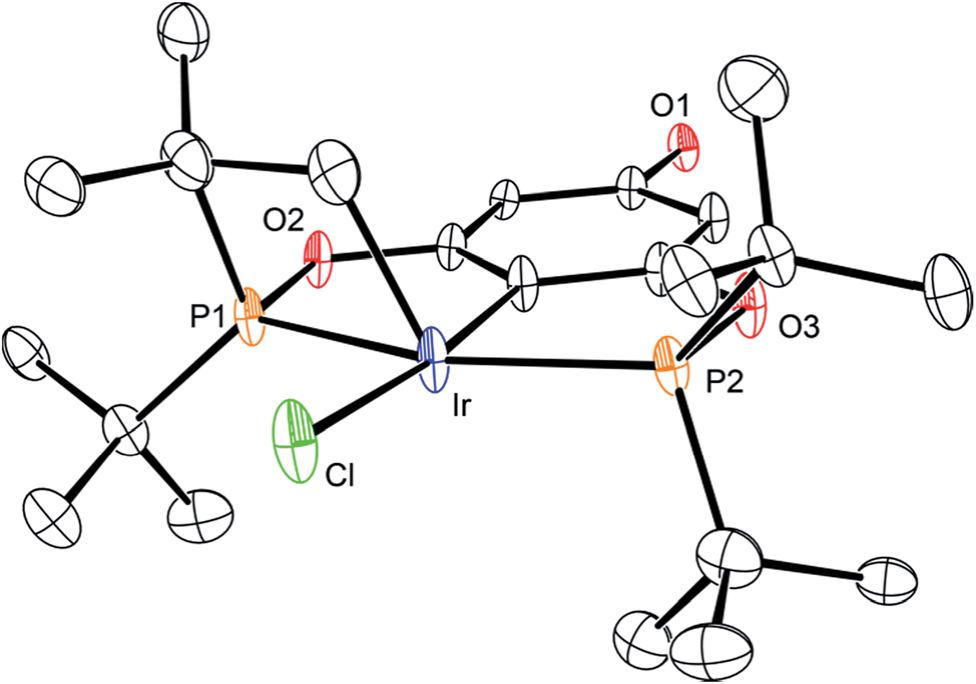
ORTEP drawing of **2**. Hydrogen atoms are omitted for clarity, and thermal ellipsoids are set to 20% probability. Only one set of disordered *tert*-butyl groups and chlorides are shown for clarity.

The synthetic route outlined in Scheme 1 was used to prepare the carboxylic acid functionalised pincer complex **3**. Firstly, the TBDMS-protected bis-phosphinite ligand (see ESI†) was reacted with [IrCl(COD)]_2_ to afford the complex **1**. Deprotection of the phenol silyl ether using Cu(ii) chloride led to the formation of the phenolic derivative **2** as the major product. At this stage we observed that one of the *tert*-butyl phosphinite substituents had reacted with the metal center to form a cyclometallated four-membered ring compound **2**. Similar complexes have been reported^13^ and their distinctive characterisation data described. The ^31^P NMR spectrum of **2** (and of the complexes obtained in the subsequent steps) shows a set of resonances (two doublets) at *δ* 160.4 and 119.3, confirming the asymmetry of the complex and the existence of two heterotopic phosphorous atoms. Notably, the hydride signal (*δ* –41.9) that characterises the complex **1** is no longer observed. Single crystal X-ray diffraction analysis confirms (Fig. 2) the structural assignment of **2**.

Etherification, using methyl chloroacetate and subsequent hydrolysis completes the synthesis of the desired carboxylic acid functionalised pincer complex **3**. Hydride iridium pincer complexes are known^14^ to activate a variety of unreactive bonds and to prevent the pincer complex from reacting in an uncontrolled fashion with the zirconium nodes or with the organic linkers in the MOF, we decided to perform SALI with the iridium–chloride complex **3** and to proceed to its activation in a subsequent step.^15^

**Scheme 2:**
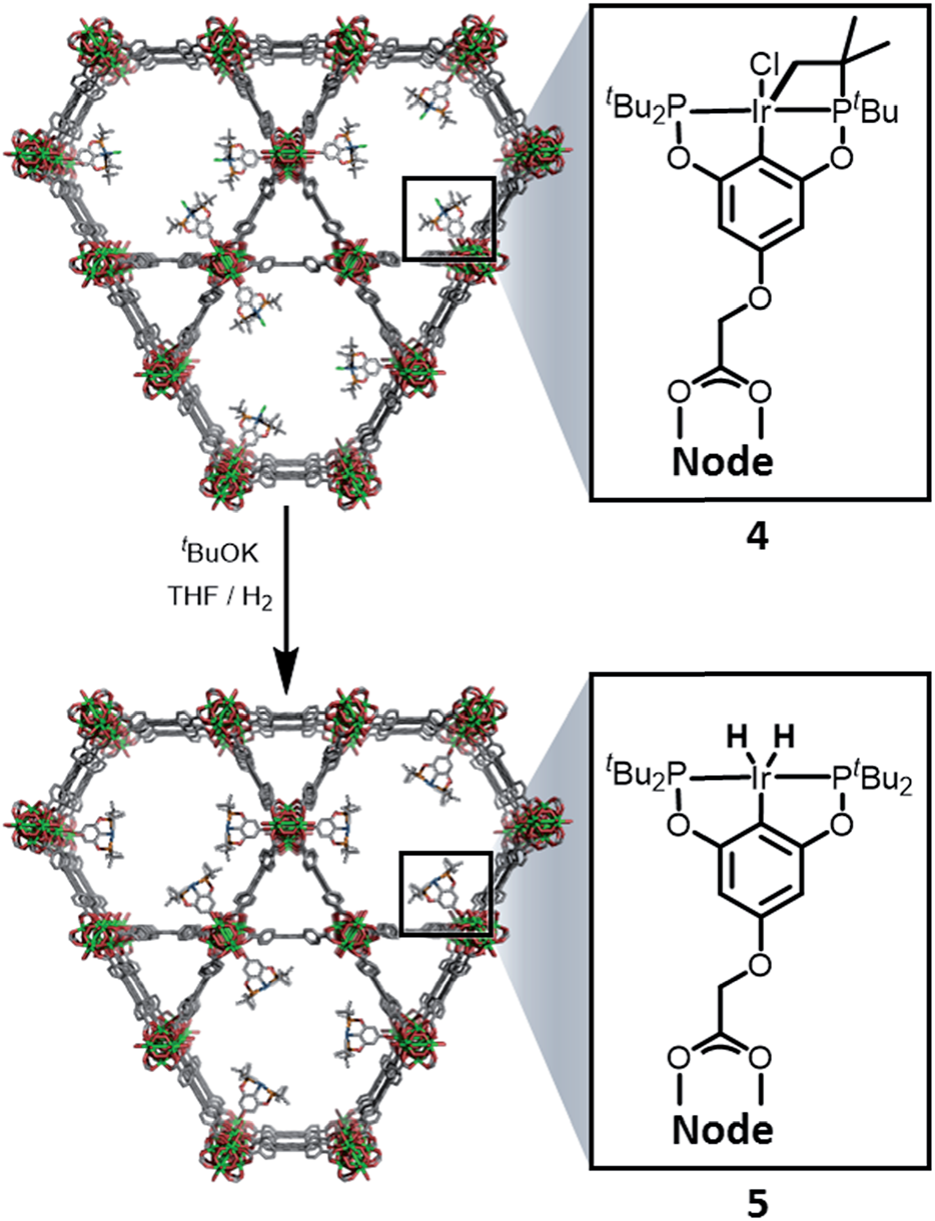
Reactivity of **4** with ^*t*^BuOK and H_2_ to give the **NU-1000** supported dihydride iridium pincer complex, **5**.

**Fig. 3:**
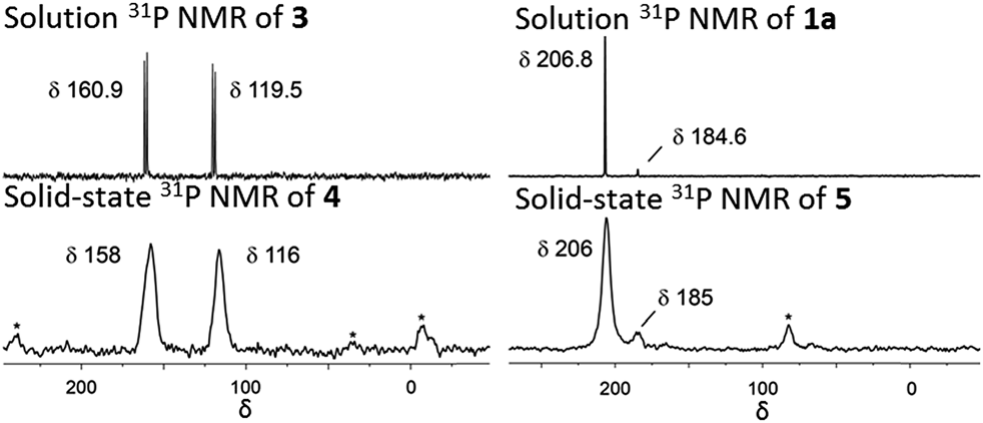
Solution phase ^31^P NMR of **3** and **1a** in C_6_D_6_ at 243 MHz. Solid-state ^31^P MAS NMR of **4** and **5** at 162 MHz. Asterisks denote spinning side bands.

**NU-1000** was suspended in a PhMe solution of **3** and allowed to react at room temperature (see ESI†). After 24 h, the MOF changed colour from yellow to orange, indicating (Scheme 2) the incorporation of the iridium complex and the formation of **4**. The solid was thoroughly washed^16^ with PhMe. Inductively coupled plasma – optical emission spectrometry (ICP-OES) of **4** showed an Ir : P : Zr ratio of 0.8 : 1.5 : 6 (in theory a 1 : 2 : 6 ratio is expected for incorporation of one iridium pincer complex per node) and confirmed the incorporation of the iridium complex into the framework with a loading of 0.8 iridium complexes per Zr_6_ node. Scanning electron microscopy-energy dispersive X-ray spectroscopy (SEM-EDS) confirms that following SALI, crystallites of **NU-1000** retain their morphology while the iridium complex is evenly distributed (see ESI†) within the crystal. The identity of the heterogenised complex was probed by solid-state ^31^P MAS NMR spectroscopy. A set of two signals at *δ* 158 and 116 was observed, in good agreement with those found (*δ* 160.9 and 119.5 see Fig. 3) for the homogenous starting material **3**. This spectroscopic evidence proves that the pincer complexes remain intact following SALI. Employing procedures previously reported^12^ for the activation of homogenous iridium pincer complexes, **4** was exposed to ^*t*^BuOK in THF under an atmosphere of H_2_ at room temperature (see ESI†). Adoption of this procedure (Scheme 2) allowed us to (i) activate the supported iridium chloride complex^17^,^18^ and (ii) form the corresponding metal hydride **5**.

Solid-state ^31^P MAS NMR spectroscopy (Fig. 3) revealed that treatment of **4** with ^*t*^BuOK and H_2_ produces a material with resonances at *δ* 206 and 185. These signals correspond^18^ to the dihydride complex (*δ* 206, major) and the tetrahydride complex (*δ* 185, minor). Additionally, infrared spectroscopy revealed (see ESI†) the appearance of a band at 2006 cm^–1^ in good agreement with the formation of a metal hydride. In addition, powder X-ray diffraction of **4** confirmed the retention of crystallinity following SALI and, more importantly, demonstrated that the framework's extended structure is maintained even after the activation step with ^*t*^BuOK to give **5** (see ESI†). Moreover, the porosity of **5** was retained as shown in Fig. S2–3.†

For comparison, we prepared (Scheme 1) the homogeneous dihydride **1a** (see ESI†) from **1**. Solution and solid-state ^31^P MAS NMR spectra showed (Fig. 3) similar resonances for **1a** and **5**, confirming the presence of analogous Ir(iii) complexes in both a homogeneous solution and a heterogeneous material.

**Fig. 4:**
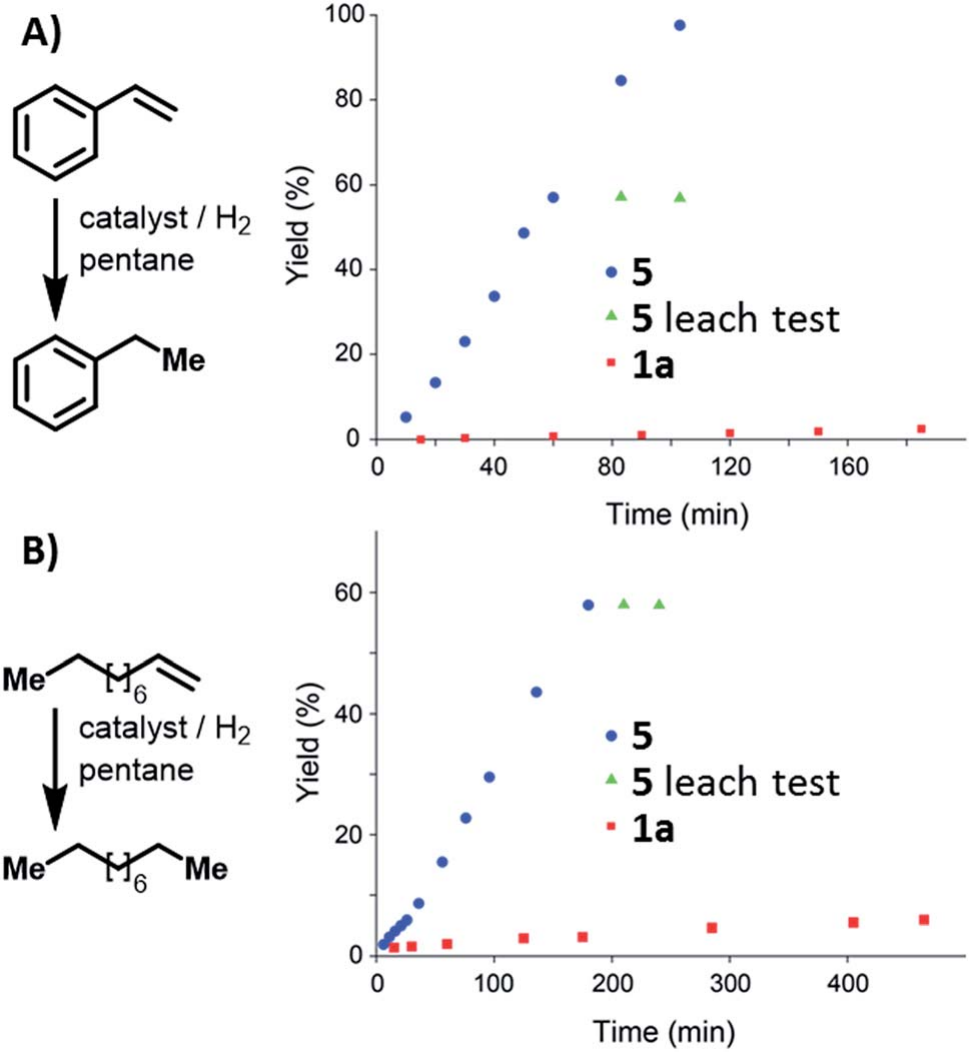
Reaction profile of styrene (**A**) and 1-decene (**B**) hydrogenation catalysed by **1a**, and **5**. Leaching tests performed using **5** indicate that the solution did not contain active catalyst.

Condensed-phase hydrogenation of 1-decene and styrene, catalysed by **1a** and **5** at 1 bar (abs.) and 23 °C, were used to examine the relative catalytic activities of the homogenous and heterogeneous Ir(iii) catalysts (see ESI†). With 1-decene as the substrate, turnover frequencies (TOFs) of 0.3 and 2.3 h^–1^ for **1a** and **5**, respectively, were calculated. Catalysis with styrene reveals TOFs of 0.1 and 7.2 h^–1^ in the case of **1a** and **5**, respectively. In both examples, **5** proved (Fig. 4) to be more active than the homogeneous catalyst **1a**.

Leaching tests on the heterogeneously catalysed reactions showed that the liquid phase is not catalytically active, confirming that the Ir(iii) pincer complex is not released into the solution.

Although a thorough explanation of such enhanced activity is not trivial, we speculate that site isolation caused by the heterogenization of the molecular complex reduces intermolecular interactions that could deactivate the catalyst or interfere in the catalytic cycle. Similar behaviour has recently been reported.8f,^19^

**Fig. 5:**
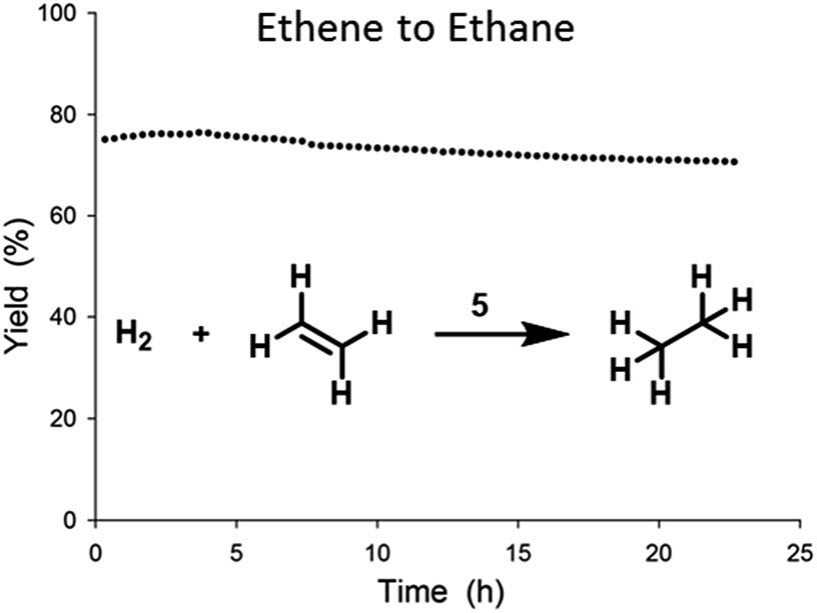
Stability test of ethene hydrogenation catalysed by **5** in flow reactor at 0.5 bar (rel.) and at 23 °C, with an ethene : hydrogen ratio of 1 : 1 and a total flow of 80 mL min^–1^.

In order to probe the activity of catalyst **5**, we tested gas-phase ethene hydrogenation in a tubular flow reactor. The TOF was determined under differential conditions at conversions below 5% (see ESI†). Catalyst **5** showed a TOF value of 0.32 s^–1^ at 50 °C, 1 bar (rel.), and with an ethene : hydrogen ratio of 1 : 1.^20^ Little deactivation was observed over time and stability tests (Fig. 5) showed only minor losses (4%) of activity over the course of 24 h.

## Conclusions

We report attaching an iridium pincer complex inside a MOF without having to synthesise the extended structure *de novo*. The complex can be activated after its incorporation to yield the active dihydrido-iridium catalyst. Despite the basic conditions required to carry out the chloride-abstraction, the crystallinity and porosity of the framework are retained and spectroscopic investigations corroborate the similarity between the solid and the related homogeneous catalyst. This observation establishes that the catalytically active site characteristic of the molecular compound has been preserved upon its heterogenization. In order to demonstrate the accessibility to the active sites, we tested this material in the hydrogenation of liquid substrates and with ethene (gas) under flow conditions – the latter being possible only with the catalyst in heterogeneous form. It is noteworthy that the heterogeneous catalyst shows enhanced activity with respect to its homogeneous counterpart and reveals stability upon prolonged use. This work serves as a proof-of-concept that well studied organometallic complexes can be installed and activated within a crystalline metal–organic framework.

## Supplementary Material

Supplementary information

Crystal structure data
