# Supplementary material for: A metal–organic framework immobilised iridium pincer complex
Source: Chem Sci. 2016 May 10;7(8):4980–4. doi: 10.1039/c6sc01376g (PMC6018438; doi:10.1039/c6sc01376g)
Supplement: Supplementary file 1 [file SC-007-C6SC01376G-s001.pdf]

Electronic Supplementary Material (ESI) for Chemical Science.
This journal is © The Royal Society of Chemistry 2016

A Metal-Organic Framework Immobilised Iridium Pincer Complex

Martino Rimoldi,[a] Akitake Nakamura,[a] Nicolaas A. Vermeulen,[a] James J. Henkelis,[a] Anthea K. Blackburn,[a] J. Joseph T. Hupp,[a] Fraser Stoddart,*[a] and Omar K. Farha*[a, b]

a) Department of Chemistry, Northwestern University, 2145 Sheridan Road, Evanston, Illinois 60208, United States

b) Department of Chemistry, Faculty of Science, King Abdulaziz University, Jeddah, Saudi Arabia.

Supplementary Information

Table of Contents

1) General Methods S3

2) Synthesis of [IrCl(*p*-HOC(O)CH_2O-PCP)] (**3**) S5

2.1) 5-(Dimethyl-tert-butylsiloxy)resorcinol S5

2.2) 5-(Dimethyl-tert-butylsiloxy)-1,3-bis(di-tert-butylphosphite)benzene — *p*-TBDMSO-PCP-H S6

2.3) [IrClH(*p*-TBDMSO-PCP)] (**1**) S6

2.4) [IrCl(*p*-HO-PCP)] (**2**) S7

2.5) [IrCl(*p*-CH_3OC(O)CH_2O-PCP)] S8

2.6) [IrCl(*p*-HOC(O)CH_2O-PCP)] (**3**) S8

3) Synthesis of [IrH_2(*p*-TBDMSO-PCP)] (**1a**) S9

4) Synthesis of **4** S10

5) Synthesis of **5** S11

6) Hydrogenation of liquid substrates catalyzed by **5** S14

7) Hydrogenation of liquid substrate catalyzed by **1a** S15

8) Hydrogenation of ethene in continuous flow catalyzed by **5** S15

9) Crystallographic characterization of [IrCl(*p*-HO-PCP)] (**2**) S17

10) References S20

1) General Methods.

Materials. All reaction vessels used in the synthesis of **3** were evacuated and filled with nitrogen three times. THF, Toluene, Dichloromethane, and DMF were supplied and directly used from dry solvent system. All the manipulations related to the synthesis of **1a**, **4**, and **5**, and the catalytic tests were carried out by means of standard Schlenk techniques (argon atmosphere) or using an argon-filled glove-box. Toluene, pentane, and THF used in the synthesis of **1a**, **4**, and **5** or in the catalytic reactions were stored over activated molecular sieves and degassed by several freeze-pump-thaw cycles. Other solvents, except those mentioned above, were used without further purification. Styrene and 1-decene were purchased from Sigma Aldrich, dried over activated molecular sieves, degassed by means of several freeze-pump-thaw cycles and stored over activated molecular sieves. Potassium *tert*-butoxide (1M solution in THF) and sodium *tert*-butoxide were purchase from Sigma Aldrich and used as received. $[IrCl(COD)]_2$ was purchased from TCI America and used as received. Deuterated solvents ($CDCl_3$, CD_3COCD_3, C_6D_6 Toluene-d_8) were purchased from Cambridge Isotope Laboratories and used without further purification. Argon (5.0), ethene (3% / Ar), and hydrogen (3% / Ar) were purchased from Airgas. Thin layer chromatography (TLC) was performed on silica gel 60 F254 (E. Merck). Column chromatography was carried out on silica gel 60F (Merck 9385, 0.040-0.063 mm). **NU-1000** was prepared according to the reported procedures.[1]

NMR measurements. Solution NMR spectra were recorded on an Agilent DD MR-400 spectrometer or a Bruker AVANCE III 600. ^{1}H and ^{13}C chemical shifts were referenced to the residual resonance of the deuterated solvent. ^{31}P NMR chemical shifts were referenced to 85 % H_3PO_4 as external standard. Solid-state ^{31}P MAS NMR spectra were recorded on a Varian 400 MHz VNMRS with a conventional triple resonance 5 mm or 3.2 mm probehead in double

resonance mode at a spinning frequency of 10.00 kHz or 20.00 kHz, respectively. The samples were filled in a glove box into a zirconia rotor. Solid-state ^{31}P chemical shifts were referenced to $(NH_4)H_2PO_4$ and the spectra baseline-corrected.

S*canning electron microscopy with energy-dispersive X-ray spectroscopy* (SEM-EDS). Samples were deposited on an aluminum stub and coated with approximately 8 nm of osmium before acquisition. The measurements were performed on a Hitachi SU8030 instrument equipped with an Oxford X-max 80 SDD EDS detector. The acquisition was performed applying an acceleration voltage of 15.0 kV and following the Lα lines for both zirconium and iridium.

Diffuse reflectance infrared fourier transform spectroscopy (DRIFTS) was performed with a Nicolet iS50 FT-IR instrument equipped with a Praying Mantis Diffuse Reflection Accessory under controlled atmosphere averaging 16 scans at the resolution of 1 cm^{-1}.

Powder X-ray diffraction (PXRD) was performed on a Rigaku ATXG diffractometer. Diffractograms were acquired over the range $1.5° < 2\Theta < 30°$ with a step width of 0.02°.

Nitrogen phsisorption. Adsorption and desorption isotherms were obtained on a Micromeritics TriStar II at 77 K. Before measurements, samples were activated on a Micromeritics Smart PrepVac at 120 °C for 24 h.

Gas chromatography analyses were performed on an Agilent 7820A CG system equipped with a FID detector and HP-5 capillary column.

Inductively coupled plasma – optical emission spectrometry (ICP-OES) was performed on a Thermo iCAP 7600 spectrometer. Samples (1 mg) were digested in sulfuric acid and hydrogen peroxide at 150 °C for 10 min using a microwave reactor.

Gas Flow catalytic measurements were conducted on a Microactivity Efficient flow reactor equipped with a stainless vertical tubular reactor. The catalyst was diluted with an inert material, typically 3 mg of catalyst were diluted with 3600 mg of silica. The reactor was handled in an argon-filled glove-box and the catalyst was deposited in the reactor over a layer of quartz wool. The temperature was controlled with a thermocouple introduced on the top of the catalyst bed. Products were identified and quantified using an on-line Agilent 7890A GC equipped with a FID detector and a GS-Alumina column (30m x 0,535mm).

2) Synthesis of [IrCl(*p*-HOC(O)CH_2O-PCP)] (3)

2.1) 5-(Dimethyl-tert-butylsiloxy)resorcinol. Imidazole (25.9 g, 381 mmol) was added to a solution of phloroglucinol (10.0 g, 79.3 mmol) and tert-butyl dimethylsilyl chloride (14.3 g, 95.2 mmol) in dry DMF (160 mL). The mixture was stirred for 18 h, diluted with 5% aqueous $NaHCO_3$, and extracted three times with ethyl acetate. The combined ethyl acetate extracts were washed with water, dried over anhydrous $MgSO_4$, and evaporated. The crude compound was purified by silica gel column chromatography using *n*-hexane/ethyl acetate (the ratio was gradually changed from 10/1 to 2/1). The solvent was removed and 5-(Dimethyl-tert-butylsiloxy)resorcinol was obtained as a colorless liquid (6.36 g, 27.9 mmol, yield 35.1 %). ^{1}H NMR (400 MHz, CD_3COCD_3, ppm): δ_H 8.07 (s, 2H), 5.99 (s, 1H), 5.89 (s, 2H), 0.96 (s, 9H), 0.18 (s, 6H).

2.2) 5-(Dimethyl-tert-butylsiloxy)-1,3-bis(di-tert-butylphosphite)benzene (*p*-TBDMSO-PCP-H). A suspension of NaH (0.466 g, 19.4 mmol) in THF (44.0 mL) was slowly added via syringe to a solution of 5-(Dimethyl-tert-butylsiloxy)resorcinol (2.00 g, 8.8 mmol) in THF (130 mL). The mixture was heated to reflux for 1 h, a solution of di-tert-butyl-chlorophophine (3.33 g, 18.4 mmol) in THF (44.0 mL) was then added via syringe, and the mixture was refluxed for additional 1 h. After evaporation of the solvent in vacuum, the residue was extracted with toluene and filtered through a pad of Celite. After removal of toluene under vacuum, the flask was heated to 80 ºC for 2 h in vacuum to remove residual amount of di-tert-butyl-chlorophophine. *p*-TBDMSO-PCP-H was obtained as white solid (3.99 g,) in 88% purity as determined by ^{31}P NMR, and used in the next reaction without further purification. ^{1}H NMR (400 MHz, $CDCl_3$, ppm): δ_H 6.57-6.53 (m, 1H), 6.31-6.28 (m, 2H), 1.13 (d, $^{3}J_{P\text{-}H}$ = 12 Hz, 36H), 0.95 (s, 9H), 0.17 (s, 6H). ^{31}P{^{1}H} NMR (161 MHz, $CDCl_3$, ppm): δ_P 152.4.

2.3) [IrClH(*p*-TBDMSO-PCP)] (1). Toluene (18.0 mL) was added to a flask containing *p*-TBDMSO-PCP-H (1.89 g, 3.2 mmol) and $[IrCl(COD)]_2$ (987 mg, 1.5 mmol). The mixture was refluxed for 14 h, cooled to room temperature, and evaporated. The residue was purified by silica gel column chromatography using *n*-hexane/ethyl acetate (the ratio was gradually changed from 40/1 until 10/1). The solvent was removed and [IrClH(*p*-TBDMSO-

PCP)] was obtained as dark red solid (1.91 g, 2.5 mmol, yield 86 %). ^{1}H NMR (400 MHz, $CDCl_3$, ppm): δ_H 6.14 (m, 2H), 1.33 (m, 36H), 0.96 (s, 9H), 0.20 (s, 6H), -41.91 (t, $^{2}J_{P\text{-}H}$ = 12 Hz, 1H). ^{31}P{^{1}H} NMR (161 MHz, $CDCl_3$, ppm): δ_P 176.44 (d, $^{2}J_{P\text{-}H}$ = 11 Hz).

2.4) [IrCl(*p*-HO-PCP)] (2). A solution of $CuCl_2$ (0.178 g, 1.3 mmol) in acetone (12.54 mL) and water (0.66 mL) was added to [IrClH(*p*-TBDMSO-PCP)] (**1**) (1.00 g, 1.3 mmol). The solution was refluxed for 24 h, cooled to room temperature, and stirred for 26 h. The cap of the flask was removed, and the solution stirred for 2 h. The solution was extracted two times with ethyl acetate and water, dried by brine, the combined ethyl acetate extracts were dried over anhydrous $MgSO_4$, filtered, and evaporated. The crude compound was purified by silica gel column chromatography using *n*-hexane/ethyl acetate (the ratio was gradually changed from 20/1 to 5/1). The solvent was removed and obtained [IrCl(*p*-HO-PCP)] (**2**) as dark red solid (571 mg, 0.89 mmol, yield 68 %). ^{1}H NMR (400 MHz, $CDCl_3$, ppm): δ_H 6.35 (s, 1H, Ar-*H*), 6.30 (s, 1H, Ar-*H*), 5.03 (s, very broad, 1H, RO*H*), 3.04 (m, 1H, Ir-C*H*), 2.03 (brs, 1H, Ir-C*H*), 1.53 (d, 3H, $^{3}J_{P\text{-}H}$ = 16 Hz, C*H₃*), 1.46 (d, 9H, $^{3}J_{P\text{-}H}$ = 16 Hz, $C(CH_3)_3$), 1.24 (d, 9H, $^{3}J_{P\text{-}H}$ = 12 Hz, $C(CH_3)_3$), 1.14 (d, 9H, $^{3}J_{P\text{-}H}$ = 16 Hz, $C(CH_3)_3$), 0.91 (d, 3H, $^{3}J_{P\text{-}H}$ = 16 Hz, CH_3). ^{31}P{^{1}H} NMR (161 MHz, $CDCl_3$, ppm): δ_P 160.35 (d, $^{2}J_{P\text{-}P}$ = 385 Hz), 119.26 (d, $^{2}J_{P\text{-}P}$ = 387 Hz). HRMS (ESI) calcd for $(M–Cl)^+$ $C_{22}H_{38}IrO_3P_2$ 605.1921; found 605.1918.

2.5) [IrCl(*p*-$CH_3OC(O)CH_2O$-PCP)]. A solution of methyl chloroacetate (68 mg, 0.63 mmol) in acetone (3.14 mL) was added to the flask containing [IrCl(*p*-HO-PCP)] (**2**) (100 mg, 0.16 mmol) and K_2CO_3 (65 mg, 0.47 mmol). The mixture was heated to reflux for 20 h, cooled to room temperature, and water was added. The aqueous layer was extracted with dichloromethane, and the organic layer was dried over anhydrous $MgSO_4$ and filtered. The crude compound was purified by silica gel column chromatography using *n*-hexane/ethyl acetate (the ratio was gradually changed from 20/1 to 5/1). The solvent was removed and [IrCl(*p*-$CH_3OC(O)CH_2O$-PCP)] was obtained as dark red solid (64 mg, 0.09 mmol, yield 58 %). ^1H NMR (400 MHz, $CDCl_3$, ppm): δ_H 6.42 (d, 1H, $J < 4$ Hz), 6.36 (d, 1H, $J = 4$ Hz), 4.62 (s, 2H), 3.82 (s, 3H), 3.04 (m, 1H), 1.99 (m, 1H), 1.54 (d, 3H, $^3J_{P\text{-}H} = 20$ Hz), 1.46 (d, 9H, $^3J_{P\text{-}H} = 16$ Hz), 1.24 (d, 9H, $^3J_{P\text{-}H} = 12$ Hz), 1.15 (d, 9H, $^3J_{P\text{-}H} = 16$ Hz), 0.91 (d, 3H, $^3J_{P\text{-}H} = 12$ Hz). $^{31}P\{^1H\}$ NMR (161 MHz, $CDCl_3$, ppm): δ_P 160.56 (d, $^2J_{P\text{-}P} = 385$ Hz), 119.46 (d, $^2J_{P\text{-}P} = 385$ Hz).

2.6) [IrCl(*p*-$HOC(O)CH_2O$-PCP)] (3). Hydrochloric acid (3N, 1.00 mL) and THF (1.00 mL) were added to [IrCl(*p*-$CH_3OC(O)CH_2O$-PCP)] (64 mg, 0.08 mmol). The mixture was heated to reflux for 14 h, cooled to room temperature, and water was added. The aqueous layer was extracted with dichloromethane, and the organic layer was dried over anhydrous $MgSO_4$ and

filtered. The crude compound was purified by silica gel column chromatography using *n*-hexane/ethyl acetate (4/1) and *n*-hexane/ethyl acetate/acetic acid (35/15/1). The solvent was removed and [IrCl(*p*-HOC(O)CH_2O-PCP)] (**3**) was obtained as dark red solid (50 mg, 0.07 mmol, yield 88 %). ^{1}H NMR (400 MHz, $CDCl_3$, ppm): δ_H 6.40 (d, 1H, $J < 4$ Hz, Ar-*H*), 6.34 (d, 1H, $J <$ 4 Hz, Ar-*H*), 4.65 (s, 2H, ArO-CH_2COOH), 3.04 (m, 1H, Ir-C*H*), 1.99 (m, 1H, Ir-C*H*), 1.54 (d, 3H, $^{3}J_{P\text{-}H}$ = 16 Hz, CH_3), 1.46 (d, 9H, $^{3}J_{P\text{-}H}$ = 16 Hz, C(CH_3)$_3$), 1.24 (d, 9H, $^{3}J_{P\text{-}H}$ = 16 Hz, C(CH_3)$_3$), 1.15 (d, 9H, $^{3}J_{P\text{-}H}$ = 16 Hz, C(CH_3)$_3$), 0.92 (d, 3H, $^{3}J_{P\text{-}H}$ = 12 Hz, CH_3). ^{31}P{^{1}H} NMR (161 MHz, $CDCl_3$, ppm): δ_P 160.77 (d, $^{2}J_{P\text{-}P}$ = 385 Hz), 119.65 (d, $^{2}J_{P\text{-}P}$ = 385 Hz). The signal associated with the proton on the carboxylic acid could not be observed.

HRMS (ESI) calcd for $(M–H)^-$ $C_{24}H_{39}ClIrO_5P_2$ 697.1589; found 697.1591.

3) Synthesis of [IrH_2(*p*-TBDMSO-PCP)] (1a).

In a typical procedure, in a glove-box [IrCl(*p*-TBDMSO-PCP)] (**1**) (33 mg, 44 μmol) and sodium *tert*-butoxide (15 mg, 156 μmol) were introduced in a 20 mL Schlenk tube. Pentane (9 mL) was added thereto under argon and the argon atmosphere replaced with hydrogen (1 bar). The solution was stirred for 2 h, the solution filtered over a glass filter and the solvent removed

in vacuum affording a red-brown solid (25 mg, 35 μmol; yield: 80 %). ^{31}P NMR: δ 206.8 (major, Ir-H_2); δ 184.6 (traces, Ir-H_4). ^{1}H NMR (C_6D_6, RT): δ 17.5 (*t*, 7.3 Hz, major, Ir-H_2); δ 8.3 (br, traces, Ir-H_4).

4) Synthesis of 4.

In a typical procedure [IrCl(*p*-HOC(O)CH_2O-PCP)] (**3**) (45 mg, 60 μmol) and **NU-1000** (90 mg, 41 μmol) were introduced in a Schlenk tube under argon equipped with a Teflon coated stirring bar and suspended in toluene (5 mL). The resulting suspension was stirred at room temperature for 24 h. The solid progressively changed color from yellow to orange with time. The solid was recovered on a glass filter and washed with toluene (10 mL) 3 times. The washed solid was dried in vacuum for 12 h. ^{31}P MAS NMR: δ 158, δ 116. ICP-OES showed an Ir / Zr_6 ratio of 0.8, indicating the incorporation of 0.8 iridium complexes per zirconium cluster.

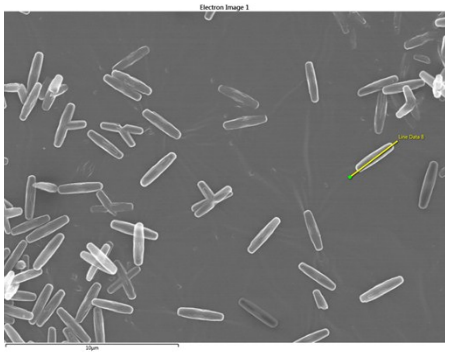

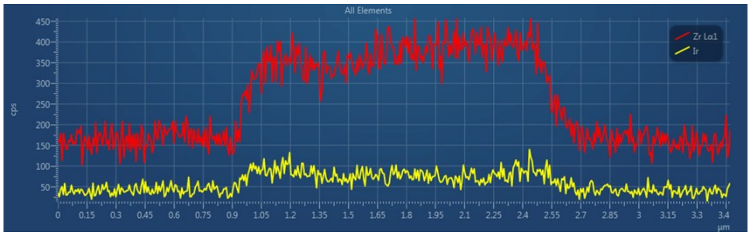

Fig. S1: Scanning electron microscopy with energy dispersive X-ray spectroscopy of **4**.

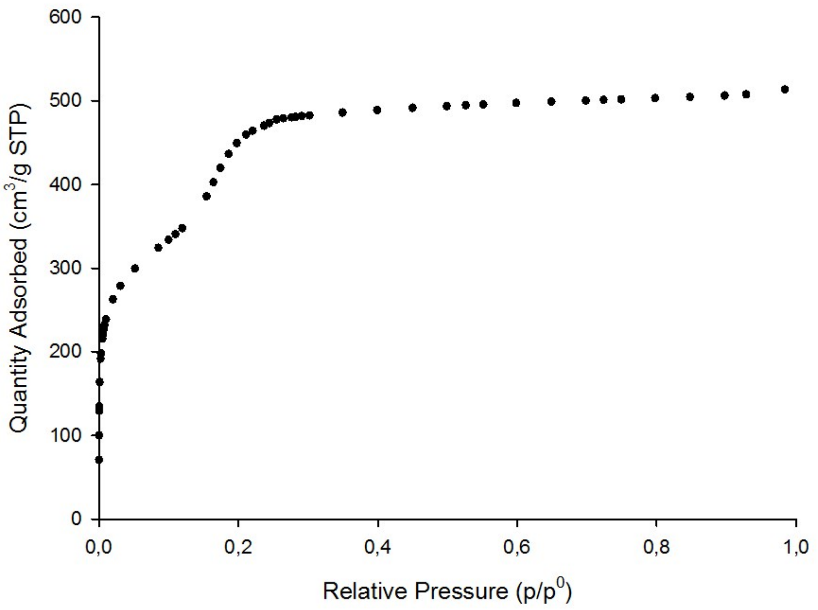

Fig. S2: Nitrogen physisorption isotherm (adsorption branch) of **4** measured at liquid nitrogen temperature. BET surface area: 1368 m^2/g.

5) Synthesis of 5.

In a typical procedure, **4** (160 mg, 44 μmol based on a loading of 0.8 iridium complexes per Zr_6) was introduced under argon in a 20 mL Schlenk tube equipped with a Teflon coated stirring bar. Potassium *tert*-butoxide (70 μL, 1M solution in THF, 70 μmol) and THF (10 mL) were added thereto. The argon atmosphere was replaced with hydrogen and the Schlenk sealed with a Teflon cap. The resulting mixture was stirred at room temperature for 24 h and the orange

suspension became light-brown. After stopping the stirring, the solid deposited on the bottom of the Schlenk tube and the solution was removed with a syringe. THF (10 mL) was added into the Schlenk and the stirred suspension was heated at 70 °C for 3 h. The warm solution was then separated and the washing procedure repeated 4 more times. The solid was collected on a glass filter, dried in vacuum for 12 h and stored in a glove-box. ^{31}P MAS NMR: δ 206 (major, Ir-H_2), δ 185 (trace, Ir-H_4). DRIFT: $\nu_{(Ir\text{-}H)}$ 2006 cm^{-1}.

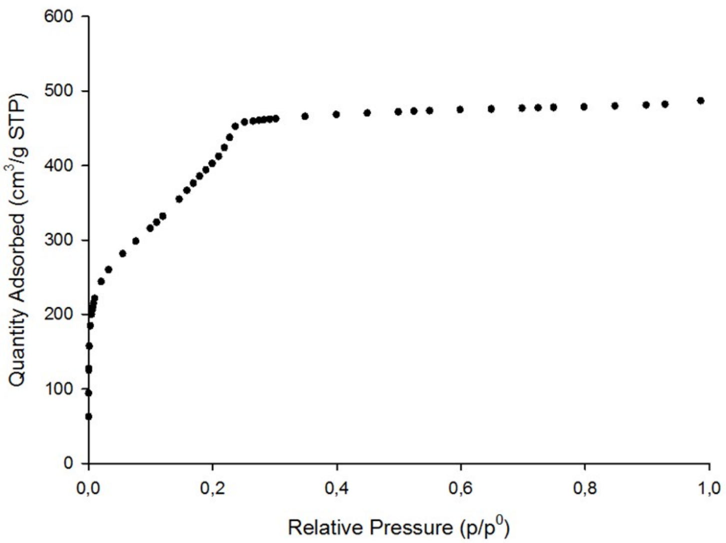

Fig. S3: Nitrogen physisorption isotherm (adsorption branch) of **5** measured at liquid nitrogen temperature. BET surface area: 1233 m^2/g.

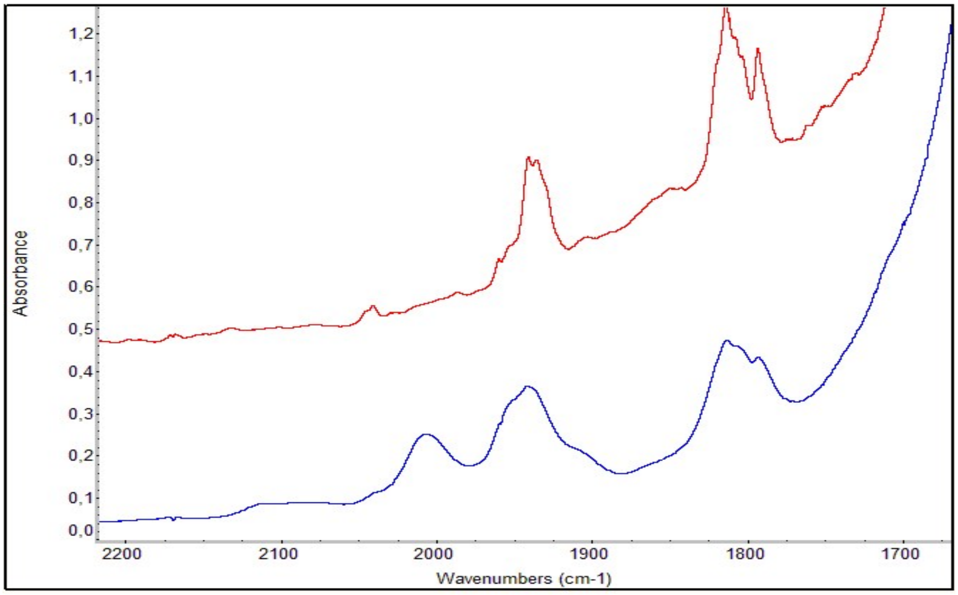

Fig. S4: Diffuse reflectance infrared spectrum of the hydride region of **5** (bottom spectrum, blue) and **NU-1000** as reference (top spectrum, red).

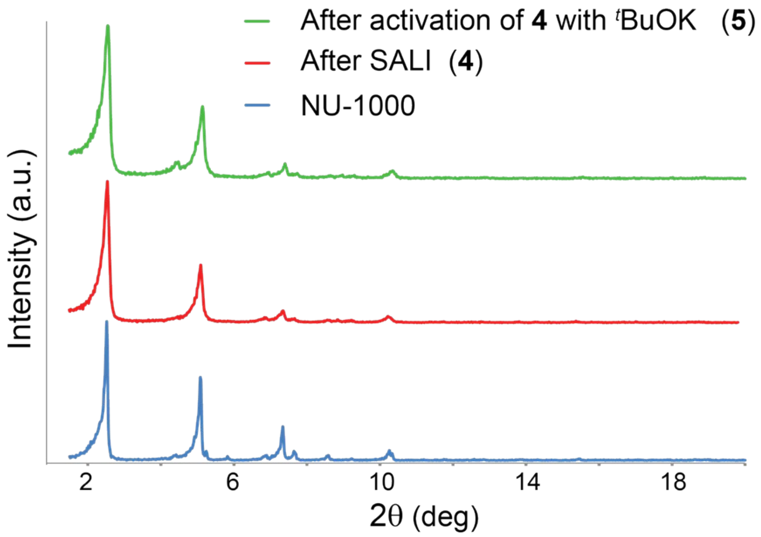

Fig. S5: Powder X-ray diffraction of **NU-1000** (bottom, blue), **4** (middle, red), and **5** (top, green).

6) Hydrogenation of liquid substrates catalyzed by 5.

Catalyst **5** (10 mg, 2.8 μmol, based on loading of 0.8 Ir / Zr_6) was introduced in a Schlenk tube equipped with a rubber septum and a Teflon coated stirring bar. Pentane (5 mL) is added thereto and the argon atmosphere replaced with hydrogen at a constant pressure of 1 bar (abs.) and a temperature of 23 °C. 1-Decene or styrene (260 or 270 μL, 2349 or 2346 μmol, respectively) were introduced into the Schlenk tube through the septum and the reaction started. Samples of the reaction mixture were periodically taken and the progress of the reaction monitored by GC. Leaching test (1-decene) was performed at approximately 60 % conversion. The stirring was interrupted and the solid catalyst deposited on the bottom of the Schlenk tube. An aliquot of the solution was transferred via a syringe in a new Schlenk tube under hydrogen and the progress of the reaction in absence of the solid catalyst monitored by GC. Split test (styrene) was performed at approximately 60 % conversion. Half of the reaction mixture was separated and introduced in a new reaction tube under hydrogen. On the transferred aliquot was performed a leaching test as described above and the remaining part of the reaction mixture was used to monitor the progress of the reaction to full conversion.

7) Hydrogenation of liquid substrate catalyzed by 1a.

Catalyst **1a** (2 mg, 2.8 μmol) was introduced in a Schlenk tube equipped with a rubber septum and a Teflon coated stirring bar. Pentane (5 mL) was added thereto and the argon atmosphere replaced with hydrogen at a constant pressure of 1 bar (abs.) and a temperature of 23 °C. 1-Decene or styrene (260 or 270 μL, 2349 or 2346 μmol, respectively) were introduced into the Schlenk tube through the septum and the reaction started. Samples of the reaction mixture were periodically taken and the progress of the reaction monitored by GC.

8) Hydrogenation of ethene in continuous flow catalyzed by 5.

TOF values were determined varying the total flow and maintaining constant the ratio between the flows of the two reagents, and at conversions below 5 %. The reactions were conducted with an ethene : hydrogen ratio of 1 : 1 and making use of 3% ethene in argon and 3% hydrogen in argon at a final concentration of 1.5 % each. TOFs were determined at 50 °C or 23 °C and 1 bar (rel.) or 0.5 bar (rel.), respectively (see Figures S6-7). The stability test was conducted at 23 °C and 0.5 bar (rel.).

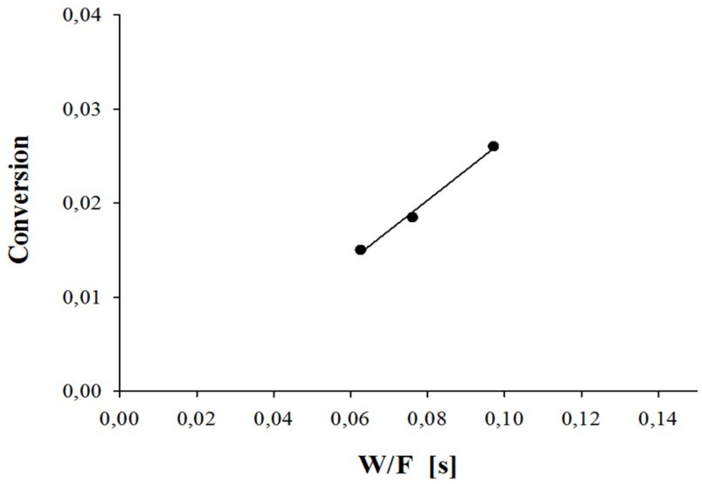

Fig. S6: TOF determination of ethene hydrogenation catalyzed by **5** at 50 °C and 1 bar (rel.). TOF: 0.321 s^{-1}.

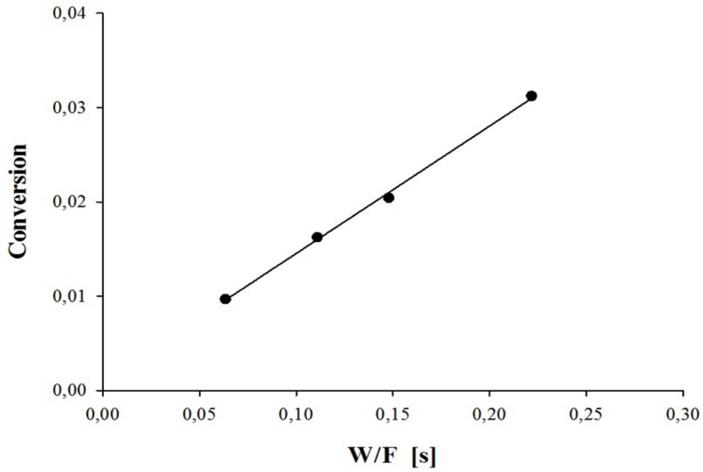

Fig. S7: TOF determination of ethene hydrogenation catalyzed by **5** at 23 °C and 0.5 bar (rel.). TOF: 0.135 s^{-1}.

9) Crystal structure of 2.

Data were collected at 100 K on a Bruker Kappa APEX2 CCD Diffractometer equipped with a CuKα microsource with Quazar or MX optics. Crystallographic data are available free of charge from the Cambridge Crystallographic Data Centre (CCDC) using via www.ccdc.cam.ac.uk/data_request/cif.

[IrCl(*p*-HO-PCP)] (**2**) was dissolved in Et_2O and the solution was poured in a 0.7 mL tube, which was placed inside a 20 mL vial containing CH_3OH. The vial was capped, and after slow evaporation of CH_3OH at -26 °C in the freezer for 1 week, deep red single crystals of [IrCl(*p*-HO-PCP)] (**2**), were obtained. ($C_{22}H_{38}ClIrO_3P_2$); red needle, 0.088 × 0.042 × 0.021 mm^3; monoclinic, space group $P2_1/n$; a = 9.8099(3), b = 20.1079(5), c = 13.9635(4) Å; $\alpha = \gamma = 90$, β = 106.852(2)°; V = 2636.1(1) $Å^3$; Z = 4; ρ_{calcd} = 1.613 g cm^{-3}; $2\theta_{max}$ = 67.573°; T = 100(2) K; 4681 reflections collected, 4512 independent, 323 parameters; μ = 12.035 mm^{-1}; R_1 = 0.0711 [$I > 2.0\sigma(I)$], wR_2 = 0.1429 (all data); CCDC deposition number 1465323. The crystal studied was comprised of two molecules of **2** disordered over one position and, as such, a reasonable amount of disorder is present in the structure, which was modeled using global bond restraints (RIGU) and distance constraints (DFIX and SADI).

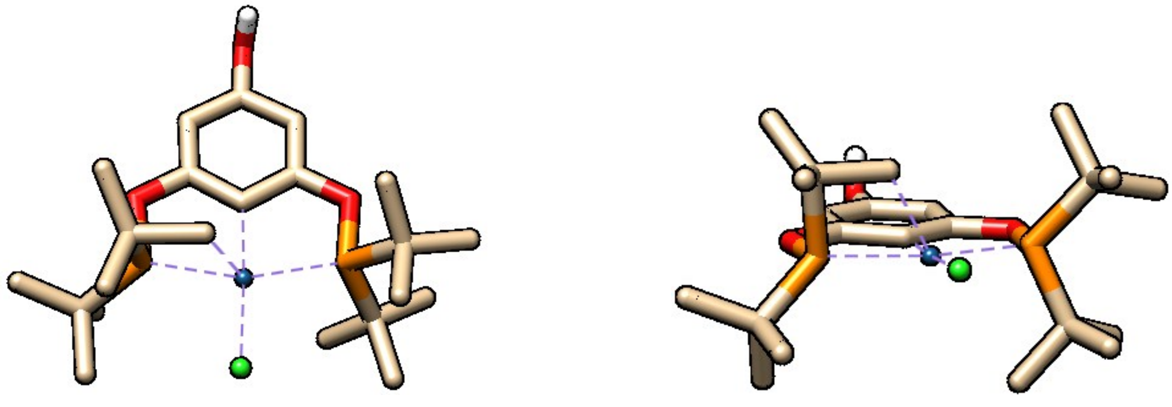

Fig. S8: different views of the structure of **2**. Hydrogen atoms are omitted for clarity

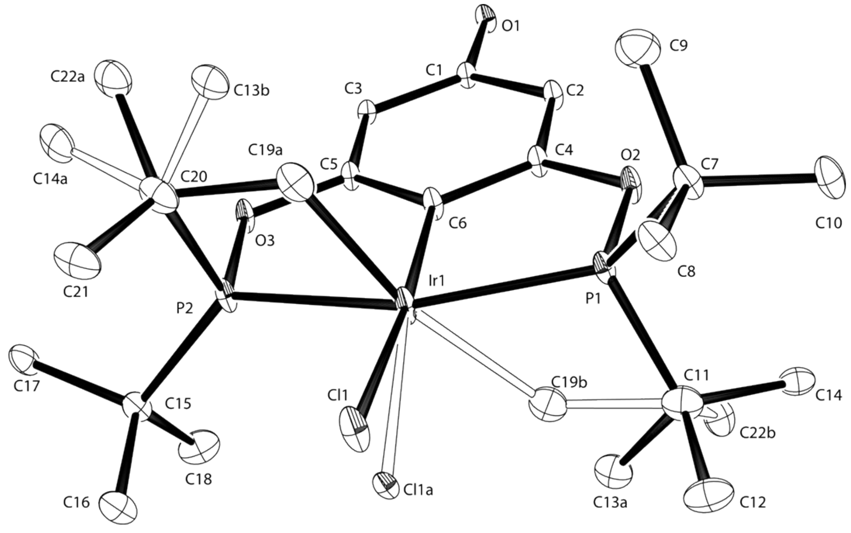

Fig. S9. ORTEP plot of **2**. The chloride ligand and the metalated *tert*-butyl unit are disordered over two positions (solid: 63%; open 37%).

Ir1 Cl1 2.420(6)	O1 C1 1.372(11)	C11 C14 1.556(13)
Ir1 Cl1A 2.520(8)	O2 C4 1.389(11)	C11 C19B 1.565(10)
Ir1 P1 2.278(3)	O3 C5 1.390(11)	C11 C22B 1.471(15)
Ir1 P2 2.267(3)	C1 C2 1.388(13)	C13B C20 1.497(15)
Ir1 C6 1.997(9)	C1 C3 1.383(13)	C14A C20 1.574(15)
Ir1 C19A 1.98(2)	C2 C4 1.389(13)	C15 C16 1.525(11)
Ir1 C19B 2.07(4)	C3 C5 1.381(13)	C15 C17 1.527(11)
P1 O2 1.650(7)	C4 C6 1.394(14)	C15 C18 1.527(12)
P1 C7 1.846(12)	C5 C6 1.381(13)	C19A C20 1.591(10)
P1 C11 1.830(15)	C7 C8 1.519(11)	C20 C21 1.541(12)
P2 O3 1.652(7)	C7 C9 1.519(13)	C20 C22A 1.499(14)
P2 C15 1.841(12)	C7 C10 1.524(11)	
P2 C20 1.799(15)	C11 C12 1.508(12)	
	C11 C13A 1.513(14)	

Table S1. Distances (Å) in **2**.

P1 Ir1 Cl1 102.23(17)	C20 P2 Ir1 95.8(5)	C12 C11 C19B 108.1(18)
P1 Ir1 Cl1A 98.0(2)	C20 P2 C15 118.2(6)	C13A C11 P1 113.1(13)
P2 Ir1 Cl1 97.86(17)	C4 O2 P1 115.1(6)	C13A C11 C14 101.9(12)
P2 Ir1 Cl1A 102.2(2)	C5 O3 P2 114.6(6)	C14 C11 P1 114.8(11)
P2 Ir1 P1 159.58(9)	O1 C1 C2 116.9(8)	C19B C11 P1 66.5(18)
C6 Ir1 Cl1 174.2(5)	O1 C1 C3 121.7(8)	C22B C11 P1 121.4(17)
C6 Ir1 Cl1A 167.7(5)	C3 C1 C2 121.3(9)	C22B C11 C12 124.5(18)
C6 Ir1 P1 80.0(3)	C1 C2 C4 118.0(9)	C22B C11 C19B 96(2)
C6 Ir1 P2 79.6(3)	C5 C3 C1 117.8(9)	C16 C15 P2 108.9(7)
C6 Ir1 C19B 89.4(11)	O2 C4 C6 118.1(8)	C16 C15 C17 110.5(10)
C19A Ir1 Cl1 84.6(6)	C2 C4 O2 118.7(9)	C16 C15 C18 109.5(11)
C19A Ir1 P1 114.7(5)	C2 C4 C6 123.1(9)	C17 C15 P2 111.3(8)
C19A Ir1 P2 63.4(5)	C3 C5 O3 118.0(8)	C18 C15 P2 107.9(9)
C19A Ir1 C6 89.7(7)	C6 C5 O3 117.8(8)	C18 C15 C17 108.6(11)
C19B Ir1 Cl1A 80.2(11)	C6 C5 C3 124.1(9)	C20 C19A Ir1 115.8(14)
C19B Ir1 P1 50.8(10)	C4 C6 Ir1 121.9(7)	C11 C19B Ir1 132(3)
C19B Ir1 P2 130.6(9)	C5 C6 Ir1 122.6(7)	C13B C20 P2 130.2(17)
O2 P1 Ir1 104.9(3)	C5 C6 C4 115.6(9)	C13B C20 C14A 102.2(16)
O2 P1 C7 101.0(4)	C8 C7 P1 108.3(8)	C13B C20 C21 106.6(14)
O2 P1 C11 101.8(5)	C8 C7 C9 108.8(11)	C14A C20 P2 101.6(17)
C7 P1 Ir1 126.8(4)	C8 C7 C10 111.7(10)	C19A C20 P2 82.8(11)
C11 P1 Ir1 107.6(5)	C9 C7 P1 106.5(9)	C21 C20 P2 112.4(10)
C11 P1 C7 111.3(6)	C9 C7 C10 108.2(11)	C21 C20 C14A 98.4(17)
O3 P2 Ir1 105.3(3)	C10 C7 P1 113.1(9)	C21 C20 C19A 109.3(12)
O3 P2 C15 100.6(4)	C12 C11 P1 114.0(10)	C22A C20 P2 119.3(13)
O3 P2 C20 103.3(5)	C12 C11 C13A 103.2(12)	C22A C20 C19A 111.3(16)
C15 P2 Ir1 130.5(4)	C12 C11 C14 108.5(14)	C22A C20 C21 116.5(14)

Table S2. Angles (°) in **2**.

10) References

1) T. C. Wang, N. A. Vermeulen, I. S. Kim, A. B. F. Martinson, J. F. Stoddart, J. T. Hupp, O. K. Farha, *Nature Protocols*, 2016, **11,** 149-162.
